# MRI-detected osteophytes of the knee: natural history and structural correlates of change

**DOI:** 10.1186/s13075-018-1734-5

**Published:** 2018-10-23

**Authors:** Zhaohua Zhu, Changhai Ding, Weiyu Han, Shuang Zheng, Tania Winzenberg, Flavia Cicuttini, Graeme Jones

**Affiliations:** 1 Clinical Research Centre, Zhujiang Hospital, Southern Medical University, Guangzhou, Guangdong China; 2 Menzies Institute for Medical Research, University of Tasmania, Hobart, TAS Australia; 3 Department of Epidemiology and Preventive Medicine, Monash University, Melbourne, VIC Australia; 4 Faculty of Health, University of Tasmania, Hobart, TAS Australia

**Keywords:** Osteoarthritis, Magnetic resonance imaging, Osteophyte, Natural history

## Abstract

**Backgroud:**

The natural history of semi-quantitative magnetic resonance imaging (MRI)-detected osteophytes (MRI-detected OPs) has not been described and it is unknown whether knee structural abnormalities can predict MRI-detected OP change over time. Thus, the aim of current study is to describe the natural history of knee MRI-detected OP, and to determine if knee structural abnormalities are associated with change of MRI-detected OP in a longitudinal study of older adults.

**Methods:**

Randomly selected older adults (*n* = 837, mean age 63 years) had MRI at baseline and 413 of them had MRI 2.6 years later to measure MRI-detected OP, cartilage defects, cartilage volume, bone marrow lesions (BMLs), meniscal extrusion, infrapatellar fat pad (IPFP) quality score/maximum area and effusion-synovitis.

**Results:**

Over 2.6 years, average MRI-detected OP score increased significantly in all compartments. The total MRI-detected OP score remained stable in 53% of participants, worsened (≥ 1-point increase) in 46% and decreased in 1%. Baseline cartilage defects (RR, 1.25–1.35), BMLs (RR, 1.16–1.17), meniscal extrusion (RR, 1.22–1.33) and IPFP quality score (RR, 1.08–1.20) site-specifically and independently predicted an increase in MRI-detected OP (*p* values all ≤ 0.05), after adjustment for covariates. Presence of IPFP abnormality was significantly associated with increased MRI-detected OPs but became non-significant after adjustment for other structural abnormalities. Total (RR, 1.27) and suprapatellar pouch effusion-synovitis (RR, 1.22) were both associated with increased MRI-detected OPs in the lateral compartment only (both *p* < 0.04).

**Conclusion:**

Knee MRI-detected OPs are common in older adults and are likely to progress. The association between baseline structural abnormalities and worsening MRI-detected OPs suggest MRI-detected OP could be a consequence of multiple knee structural abnormalities.

## Background

Osteoarthritis (OA) is a major contributor to overall disability in Western populations. Osteophytes (OPs) have long been viewed as a defining structural feature of knee OA [1] and a fundamental sign of disease incidence and progression [2]. They are associated with radiographic joint space narrowing, subchondral sclerosis, and cartilage defects in both tibiofemoral and patellofemoral compartments [3, 4]. The size and extent of OP formation is routinely used for classifying the stage of OA [5].

Conventional radiographs remain the gold standard for assessment of OPs [6], but the association between presence of knee OPs on radiographs and symptoms is poor [7]. Two-dimensional conventional radiographs may miss the extent and size of OPs [8]. On the other hand, magnetic resonance imaging (MRI) is a non-invasive multiplanar tomographic modality that can assess many knee osteoarthritic changes [9]. Studies suggest that MRI can assess OPs with much greater sensitivity than radiographs and in locations that are not easily visualised by conventional radiography due to its ability to provide three-dimensional information [10–12]. Our recent study reported that MRI-detected OPs are highly prevalent in older adults (85% vs 10% radiographic OPs prevalence) and can independently and site-specifically predict increases in cartilage defects, BMLs and loss of cartilage volume, and worsening knee pain over time, suggesting MRI-detected OPs are clinically relevant [13].

As far as we know, there are seldom longitudinal studies [14–16] investigating the relationship between MRI-detected OPs and clinical changes of OA. Sowers et al. found that large MRI-detected OPs were associated with increased odds of knee pain and reduced physical function [14]. Hakky et al. measured OP volume using MRI and observed significant positive correlation between OP volume and cartilage thickness loss [16]. In the latest study, MRI-detected OPs in a group of patients with end-stage OA scored using the Whole-Organ Magnetic Resonance Imaging Score (WORMS) grading system, those with a MRI-detected OP score of more than 30 have about threefold higher risk of undergoing total knee arthroplasty [15]. Based on the Chingford study, the natural history of radiographic OA is that of very slow progression [17]. However, the natural history of semi-quantitative MRI-detected OPs has not been described and it is unknown whether knee structural abnormalities, including cartilage defects, bone marrow lesions (BMLs), meniscal extrusion, infrapatellar fat pad (IPFP), and effusion-synovitis, can predict MRI-detected OP change over time. Hence, the aims of this study were to describe the natural history of knee MRI-detected OP, and to determine if knee structural abnormalities are associated with change of MRI-detected OP in a longitudinal study of older adults.

## Methods

### Participants

This study was from the Tasmania Older Adult Cohort (TASOAC) Study, a population-based, ongoing, prospective longitudinal cohort study which was designed to identify the genetic, environmental, and biochemical factors associated with the development and progression of OA at multiple sites. Participants between 50 and 80 years old were randomly selected from the electoral roll in Southern Tasmania (population 229,000) with an equal number of men and women (response rate 57%). Participants were excluded if they were institutionalized or had contraindications to MRI. The Southern Tasmania Health and Medical Human Research Ethics Committee approved the study, and written informed consent was obtained from all participants. Baseline examinations were made between February 2002 and September 2004, and follow-up measures were made at approximately 2.6 years later. This study consisted of 837 participants who had both knee MRI and radiographic scans at baseline.

### Anthropometrics

Weight was measured using electronic scales (nearest 0.1 kg), with shoes, socks and bulky clothing removed. Height was measured using a stadiometer (nearest 0.1 cm), with shoes, socks and headgear removed. Body mass index (BMI) was calculated using height and weight (kg/m^2^).

### Magnetic resonance imaging

MRI scans of the right knees were performed on two occasions and imaged in the sagittal plane on a 1.5-T whole body magnetic resonance unit (Picker, Cleveland, OH, USA) using a commercial transmit-receive extremity coil. The image sequences used are listed as follows: (1) T1-weighted fat-saturation 3D gradient recall acquisition in the steady state; flip angle 30°; repetition time 31 ms; echo time 6.71 ms; field of view 16 cm; 60 partitions; 512 × 512 matrix; acquisition time 11 min 56 s; one acquisition. Sagittal images were obtained at a partition thickness of 1.5 mm and an in-plane resolution of 0.31 × 0.31 (512 × 512 pixels); (2) T2-weighted fat-saturation 3D fast spin echo, flip angle 90, repetition time 3067 ms, echo time 112 ms, field of view 16 cm, 15 partitions, 228 × 256-pixel matrix; sagittal images were obtained at a partition thickness of 4 mm with a between-slices gap of 0.5 to 1.0 mm. The image database was transferred to an independent computer workstation using the software program Osirix (University of Geneva, Geneva, Switzerland) as previously described [18, 19].

### MRI-detected osteophytes

**Fig. 1:**
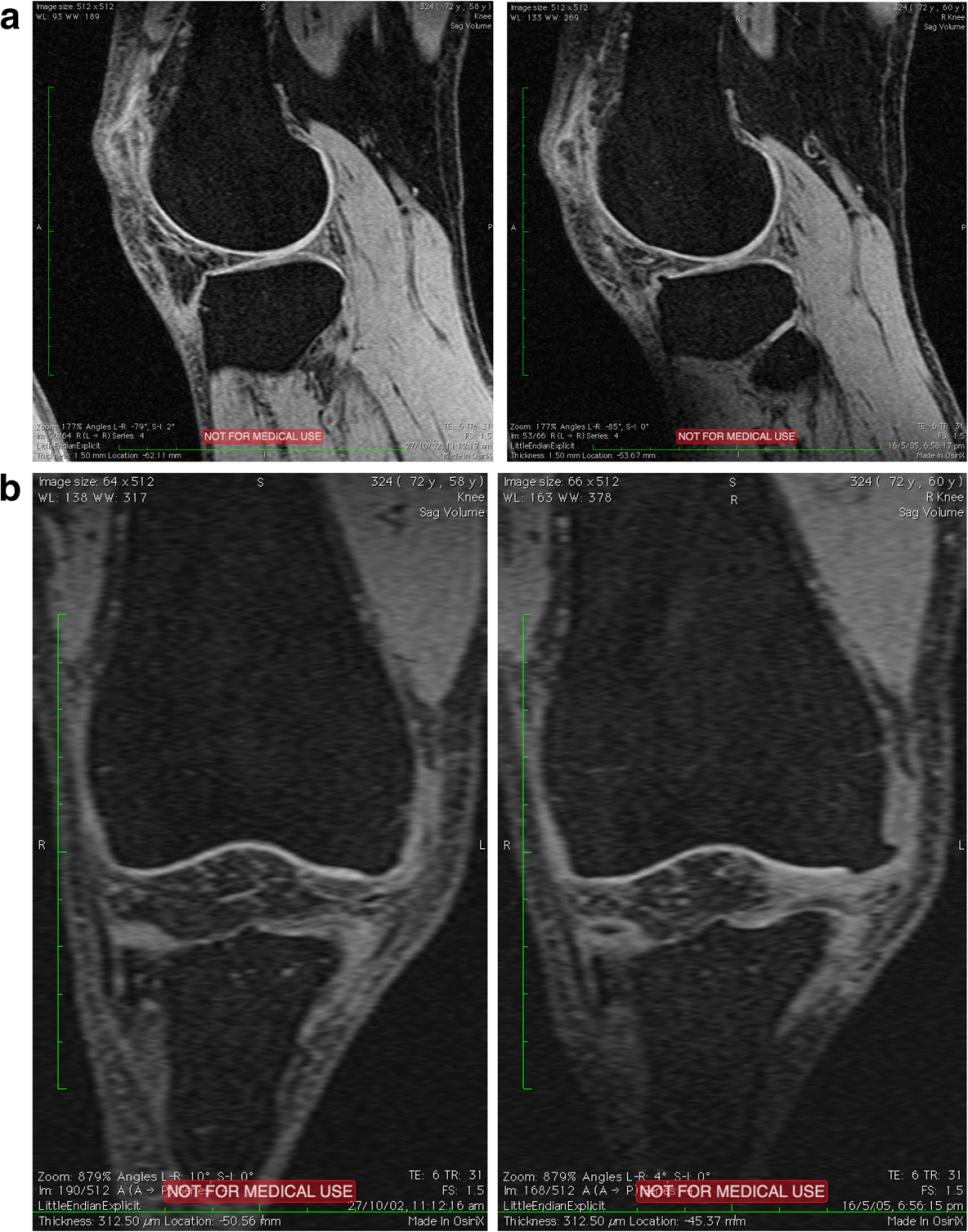
Sample images show magnetic resonance imaging (MRI)-detected osteophyte (OP) progression. **a** Tibial MRI-detected OP increases from baseline to follow up (wider arrow indicates follow-up MRI-detected OP). **b** Femoral MRI-detected OP increases from baseline to follow up (wider arrow indicates follow-up MRI-detected OP)

MRI-detected OPs were measured by ZZ using a combination of Whole-Organ Magnetic Resonance Imaging Score (WORMS) and the Knee Osteoarthritis Scoring System (KOSS) [9, 20]. OPs were defined as focal bony excrescences, seen on sagittal, axial or coronal images, extending from a cortical surface. Size was measured from the base (distinguished from that of adjacent articular cartilage with a normal MRI appearance) to the tip of the OP [11] at each of the following 14 sites: the anterior (a), central weight bearing (c) and posterior (p) margins of the femoral condyles and tibial plateaus, and the medial (M) and lateral (L) margins of the patella [20] (Fig. 1). OPs were graded as follows: grade 0, absent; grade 1, minimal (< 3 mm high); grade 2, moderate (3–5 mm); grade 3, severe (> 5 mm) [9]. The sum total score of each individual site in the relevant compartment (or whole knee) was regarded as the OP score in that compartment (or whole knee). MRI-detected OP score ≥ 1 was considered as OP present. An increase in the MRI-detected OP score from baseline to follow up at any site was defined as a change of ≥ 1 at the site. MRI-detected OPs were remeasured by ZZ and WH in 40 randomly selected participants, with a 4-week interval, to calculate intra-observer and inter-observer reliabilities. Intra-observer reliability (expressed as intraclass correlation coefficients, ICCs) was 0.94–0.97 and inter-observer reliability was 0.90–0.96 [21].

### Cartilage defects

Cartilage defects were graded by CD at the medial tibial, lateral tibial, medial femoral, lateral femoral and patellar regions as previously described [22] as follows: grade 0, normal cartilage; grade 1, focal blistering and low-signal intensity change with an intact surface and bottom; grade 2, irregularities on the surface or bottom and loss of thickness < 50%; grade 4, full-thickness cartilage loss with exposure of subchondral bone [23]. The highest score of each individual site in the relevant compartment (or whole knee) was regarded as the cartilage defect score in that compartment (or whole knee). The presence of cartilage defects was defined as a cartilage defect score ≥ 2 at any site. An increase in cartilage defects was defined as a change in cartilage defects ≥ 1. Intra-observer reliability was 0.89–0.94 and inter-observer reliability was 0.85–0.93 [22].

### Cartilage volume

Knee cartilage volume was measured on T1-weighted images by a single trained observer at baseline as previously described [23, 24]. The volumes of individual cartilage plates (medial tibial, lateral tibial, medial femoral, lateral femoral and patellar) were isolated from the total volume by manually drawing disarticulation contours around the cartilage boundaries on a section by section basis. These data were resampled by means of bilinear and cubic interpolation (area of 312 × 312) μm and 1.5 mm thickness, continuous sections) for the final 3D rendering. Changes in cartilage volume were calculated as:

Percentage change per annum = [(Follow-up volume – Baseline volume)/Baseline cartilage volume]/Time between 2 scans in years × 100.

The coefficients of variation (CVs) for cartilage volume measures were 2.1% to 2.6% [23, 24].

### Bone marrow lesions

Subchondral bone marrow lesions (BMLs) were defined as a discrete area of increased signal adjacent to the subcortical bone on T2-weighted MRI and were scored at the medial tibial, lateral tibial, medial femoral, lateral femoral, medial patellar and lateral patellar regions using a modified version of WORMS: grade 0, absence of BML; grade 1, area < 25% of the region; grade 2, area between 25% and 50% of the region; grade 3, area > 50% of the region [20]. The highest score of each individual site in the relevant compartment (or whole knee) was regarded as the BML score in that compartment (or whole knee). An increase in BMLs was defined as a change in BMLs ≥ 1. The inter-observer reliability of this BML scoring system was assessed by randomly selecting 40 subjects with BMLs and having their MRI scans re-read by another observer. The ICCs for inter-observer reliability were also excellent (0.73–0.95) [25, 26].

### Meniscal extrusion

Meniscal extrusion was assessed by a trained observer on T1-weighted MRI as previously described [27]. The proportion of the menisci affected by full extrusion was scored on the medial and lateral edges of the tibiofemoral joint space using a semi-quantitative scale. The extent of meniscal extrusion, not including the osteophytes, was evaluated for the anterior, middle and posterior horns of the menisci with 0, no extrusion; 1, partial extrusion and 2, complete extrusion with no contact with the joint space (severe). The intra-observer and inter-observer correlation coefficient ranged from 0.85 to 0.92 [28].

### Infrapatellar fat pad

The infrapatellar fat pad (IPFP) was measured both semi-quantitatively and quantitatively. IPFP maximum area was measured by manually drawing disarticulation contours around the IPFP boundaries on a secton-by-section T2-weighted MR image, using the software program Osiris (University of Geneva). The maximal area was selected to represent the IPFP size. One observer graded the IPFP area in all MRI examinations. The intra-class correlation coefficient was 0.96 for intra-observer reliability and was 0.92 for inter-observer reliability [29]. The IPFP quality score was assessed semi-quantitatively according to its signal intensity alteration, which was defined as discrete areas of increased signal within IPFP: grade 0, no signal intensity alteration; grade 1, < 10% of the region has altered signal intensity; grade 2, 10–20% of the region; grade 3, > 20% of the region. Intra-observer and inter-observer reliability was high (intra-class correlation coefficient of 0.90 and inter-class correlation coefficient of 0.89, respectively) [30]. Presence of IPFP abnormality was defined as a signal intensity alteration score ≥ 1.

### Effusion-synovitis

Knee effusion-synovitis at baseline was defined as the presence of intra-articular fluid-equivalent signal on T2-weighted MRI. We measured effusion-synovitis in the following subregions: (1) suprapatellar pouch, extending superiorly from the upper surface of the femur; (2) central portion, lying between the central femoral and tibia condyles, around the ligaments and menisci; (3) posterior femoral recess, lying behind the posterior portion of each femoral condyle and the deep surface of the lateral and medial heads of the gastrocnemius; (4) subpopliteal recess, lying posteriorly between the lateral meniscus and the popliteal tendon. Effusion-synovitis in each subregion was scored from 0 to 3 in terms of the estimated maximal distention of the synovial cavity: 0, normal; 1 < 33% of maximum potential distention; 2, 33–66% of maximum potential distention; 3, > 66% of maximum potential distention [20]. The two independent observers scored images blinded to participant information. The intra-class reliability and the inter-class reliability in different subregions was 0.63–0.79 [31].

### Statistical analysis

Student’s *t* test or the chi-square (χ^2^) test was used to compare means or proportions between participants with or without an increase in total knee MRI-detected OPs. The paired *t* test was used to compare means between baseline and follow-up MRI-detected OPs in different compartments. Crude and adjusted log binominal regression was used to examine the longitudinal associations between increases in MRI-detected OPs (dependent variable), and baseline knee BMLs, cartilage defects, meniscal extrusion, effusion-synovitis and IPFP (independent variables), with age, sex, BMI and all the structural abnormalities as covariates. All statistical analyses were performed in Stata version 12.0 for Windows (StataCorp, College Station, TX, USA) [12]. A *p* value < 0.05 (two-tailed) or a 95% confidence interval (CI) not including a value of 1.00 was considered statistically significant.

## Results

### Characteristics of the study population

A total of 1099 participants aged between 51 and 81 (mean 63) years were recruited to the TASOAC study of whom 837 had radiographs and MRI scans taken at baseline. The current study consists of a sample of 413 participants who had completed MRI scans at baseline and follow up. MRI scans were discontinued after this follow up due to decommissioning of the scanner. As reported previously [13], participants who did not complete follow-up MRI measures were similar to the remainder of the cohort in terms of demographics, smoking status, cartilage defects, BMLs, cartilage volume and radiographic OA at baseline. The characteristics of participants grouped by whether they had an increase or no increase in total knee MRI-detected OPs over 2.6 years of follow up are shown in Table 1. Participants with an increase in MRI-detected OPs over 2.6 years had significantly higher baseline BMI, MRI-detected OP score, cartilage defect score, BML score, meniscal extrusion score, total and suprapatellar effusion-synovitis score and IPFP quality score.

**Table 1:** Characteristics of participants at baseline

	Stable or decreased OPs	Increased OPs	*p*
	*N* = 222	*N* = 191	
Age (years)	62.2 ± 7.3	62.9 ± 7.0	0.30
Female (%)	53	47	0.22
BMI (kg/m^2^)	**26.9 ± 3.9**	**28.5 ± 4.9**	**< 0.01**
Baseline total cartilage defect score (0–4)	**1.48 ± 0.73**	**2.13 ± 1.03**	**< 0.01**
Baseline total BML score (0–3)	**0.33 ± 0.56**	**0.59 ± 0.78**	**< 0.01**
Baseline total tibial CV (ml)	5.11 ± 1.22	5.08 ± 1.13	0.80
Baseline meniscal extrusion score (0–2)	**0.13 ± 0.36**	**0.27 ± 0.51**	**< 0.01**
Total effusion-synovitis score (0–3)	**1.90 ± 0.67**	**2.08 ± 0.81**	**0.01**
Suprapatellar effusion-synovitis score (0–3)	**1.53 ± 0.64**	**1.76 ± 0.84**	**0.01**
Central portion effusion-synovitis score (0–3)	1.56 ± 0.71	1.65 ± 0.77	0.19
Posterior femoral recess effusion-synovitis score (0–3)	0.66 ± 0.79	0.76 ± 0.78	0.14
Subpopliteal recess effusion-synovitis score (0–3)	**0.60 ± 0.79**	**0.78 ± 0.94**	**0.04**
IPFP area (cm^2^)	7.6 ± 1.2	7.7 ± 1.3	0.18
Presence of IPFP abnormality (%)	75%	82%	0.10
IPFP quality score (0–6)	**1.45 ± 1.21**	**2.12 ± 1.64**	**< 0.01**
Baseline total OP score (0–36)	**2.80 ± 3.45**	**7.62 ± 7.70**	**< 0.01**

The two-tailed *t* test was used to examine differences between means, and the χ2 test was used for proportions (percentages). Significant differences are shown in bold. Mean ± SD except for percentages

*BMI* body mass index, *BM*: bone marrow lesion, *OP* osteophyte, *CV* cartilage volume, *IPFP* infrapatellar fat pad

### Natural history of MRI-detected OPs

**Fig. 2:**
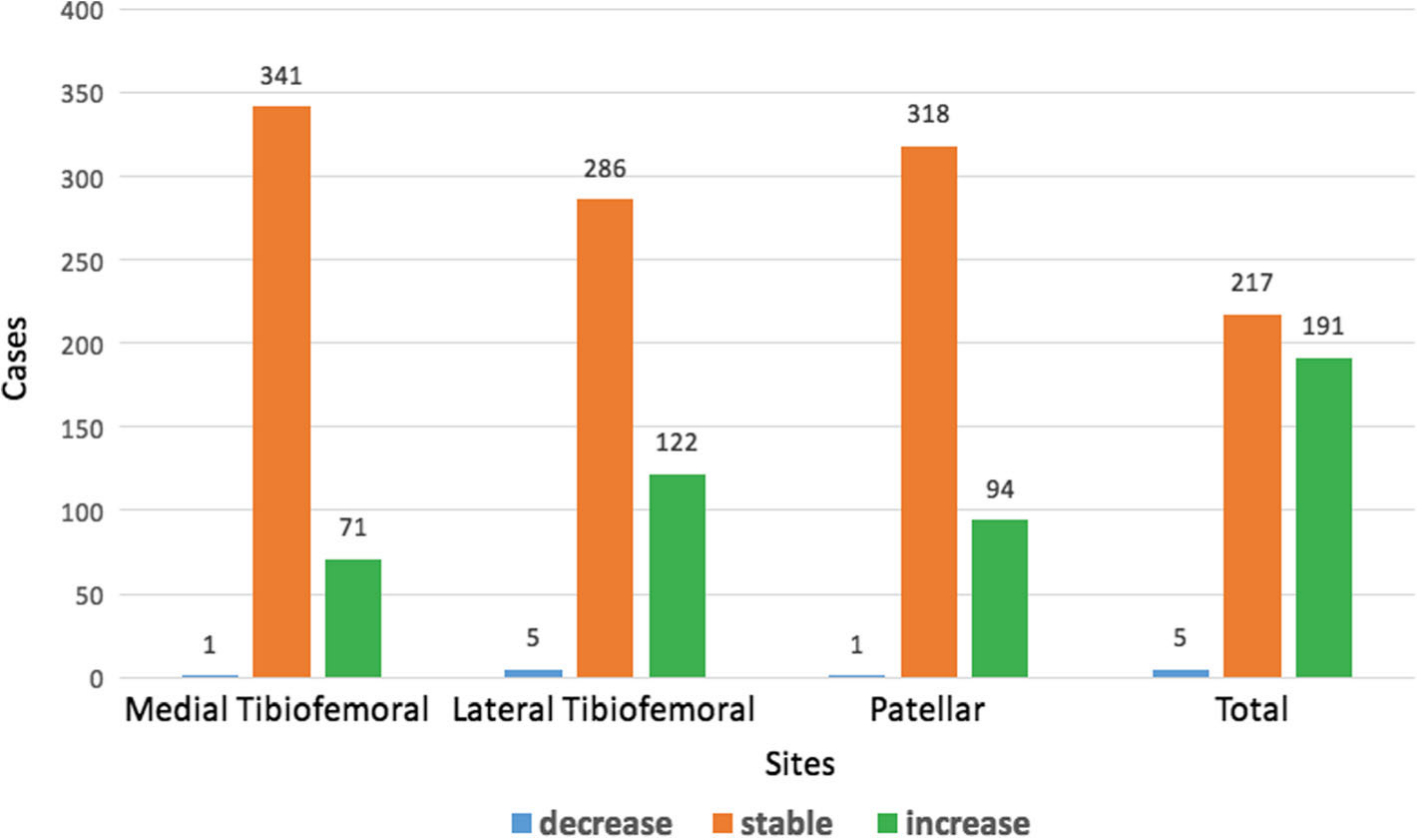
Change in magnetic resonance imaging (MRI)-detected osteophyte (OP) scores by site over 2.6 years. Total score was calculated by summing medial tibiofemoral (tibfem), lateral tibiofemoral and patellar scores

**Fig. 3:**
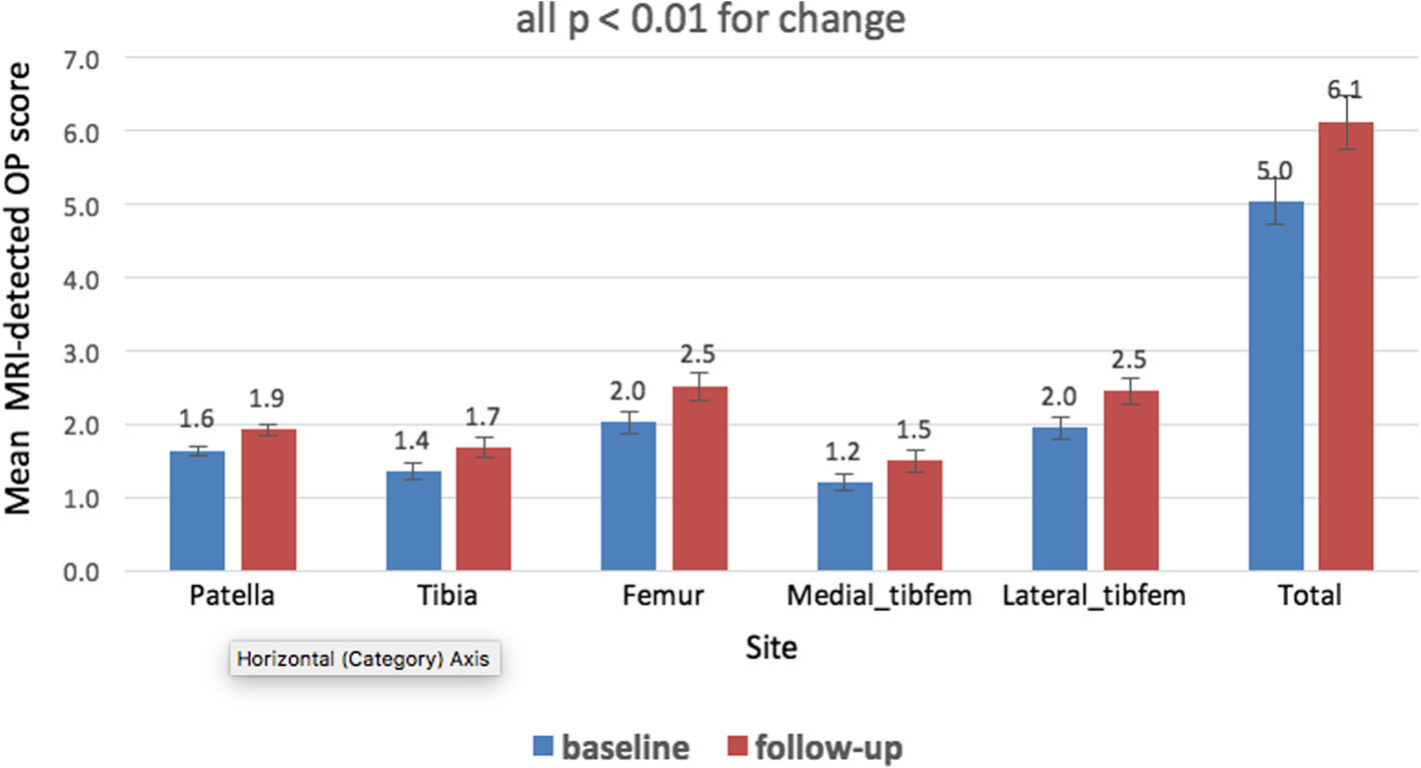
Average magnetic resonance imaging (MRI)-detected osteophyte (OP) scores increased significantly in all compartments over 2.6 years

The changes in MRI-detected OP scores by site over 2.6 years are presented in Fig. 2: 413 participants had completed follow-up MRI scans, 83%, 69%, 77%, and 53% of these participants had stable MRI-detected OP scores in the medial tibiofemoral, lateral tibiofemoral, patellar and total knee, respectively. Of these participants, 17%, 30%, 23% and 46% had increased MRI-detected OP scores in the medial tibiofemoral, lateral tibiofemoral, patellar and total knee, respectively. Of the 413 participants (86%) who completed follow up, 356 had knee MRI-detected OPs at baseline. Of these participants, 48% persisted in total MRI-detected OP size, 51% increased in total MRI-detected OP size, and only 1% decreased in total MRI-detected OP size. At baseline, MRI-detected OPs were absent in a total of 57 participants, of whom 11 (19%) developed new OPs. Over 2.6 years, the average MRI-detected OP scores in all compartments increased significantly (Fig. 3).

### Factors associated with an increase in MRI-detected OPs

Table 2 gives the association between baseline BMLs, cartilage defects, meniscal extrusion and increases in MRI-detected OPs over 2.6 years. Higher baseline BML scores and meniscal extrusion scores in medial tibiofemoral, lateral tibiofemoral and total compartments were significantly associated with increased MRI-detected OPs in the corresponding compartments, after adjustment for age, sex and BMI. These associations remained after further adjustment for baseline MRI-detected OPs and other structural abnormalities, except for the associations with BMLs in the lateral tibiofemoral and patellar compartments. Higher baseline cartilage defect score in the medial tibiofemoral, lateral tibiofemoral and total compartments was significantly associated with increased MRI-detected OPs in the corresponding compartments, after adjustment for age, sex and BMI, and remained significant after further adjustment for baseline MRI-detected OPs and other structural abnormalities.

**Table 2:** Site-specific association with increase in MRI-detected OPs over 2.6 years

	Multivariable*	Multivariable**
	RR (95% CI)	RR (95% CI)
Increase in MRI-detected OPs (yes or no)		
Medial BMLs	**1.37 (1.19, 1.58)**	**1.17 (1.02, 1.35)**
Lateral BMLs	**1.81 (1.11, 2.97)**	1.11 (0.84, 2.40)
Patellar BMLs	**1.16 (1.02, 1.33)**	1.14 (0.94, 1.37)
Total BMLs	**1.30 (1.19, 1.42)**	**1.16 (1.03, 1.31)**
Medial cartilage defects	**1.39 (1.25, 1.54)**	**1.30 (1.16, 1.45)**
Lateral cartilage defects	**1.42 (1.26, 1.61)**	**1.35 (1.21, 1.51)**
Patellar cartilage defects	**1.34 (1.22, 1.47)**	**1.25 (1.13, 1.38)**
Total cartilage defects	**2.19 (1.71, 2.80)**	**1.33 (1.20, 1.46)**
Medial meniscal extrusion	**1.39 (1.12, 1.71)**	**1.24 (1.00, 1.55)**
Lateral meniscal extrusion	**1.50 (1.27, 1.79)**	**1.33 (1.14, 1.56)**
Total meniscal extrusion	**1.40 (1.17, 1.69)**	**1.22 (1.01, 1.47)**

Dependent variable: increases in magnetic resonance imaging (MRI)-detected osteophytes (OPs) (yes or no) in the same compartment as each exposure. Independent variables: baseline bone marrow lesions (BMLs), cartilage defects, meniscal extrusion (per grade)

*Adjusted for age, sex and body mass index

**Further adjusted for other structural abnormalities and baseline OP scores. Bold denotes statistical significance

Table 3 shows the associations between baseline effusion-synovitis, IPFP and increased MRI-detected OPs over 2.6 years. Baseline total and suprapatellar effusion-synovitis scores were significantly associated with increases in total MRI-detected OP after adjustment for age, sex and BMI, but this did not persist after further adjustment for baseline OPs, BMLs and cartilage defects. Baseline effusion-synovitis score was not associated with increased medial MRI-detected OPs. In the lateral tibiofemoral compartment, baseline total, suprapatellar and central portion effusion-synovitis were significantly associated with increased MRI-detected OPs after adjustment for age, sex and BMI. The associations with total effusion-synovitis and suprapatellar effusion-synovitis but not central portion effusion-synovitis remained after further adjustment.

**Table 3:** Associations between baseline effusion-synovitis/IPFP and increased MRI-detected OPs over 2.6 years

	Multivariable*	Multivariable**
	RR (95% CI)	RR (95% CI)
Increased total MRI-detected OP (yes or no)		
Total effusion-synovitis	**1.23 (1.05, 1.45)**	1.08 (0.94, 1.24)
Suprapatellar effusion-synovitis	**1.25 (1.09, 1.45)**	1.08 (0.92, 1.64)
Central portion effusion-synovitis	1.11 (0.96, 1.29)	1.06 (0.94, 1.20)
Posterior femoral recess effusion-synovitis	1.14 (0.97, 1.33)	1.05 (0.93, 1.19)
Subpopliteal recess effusion-synovitis	**1.15 (1.03, 1.29)**	1.07 (0.97, 1.18)
IPFP quality score (0–6)	**1.17 (1.10, 1.24)**	**1.08 (1.01, 1.15)**
IPFP maximum area (cm^2^)	1.04 (0.93, 1.16)	1.03 (0.93, 1.14)
IPFP abnormal change (yes/no)	1.21 (0.91, 1.60)	0.91 (0.69, 1.22)
Increased medial MRI-detected OP (yes or no)		
Total effusion-synovitis	1.25 (0.91, 1.73)	1.13 (0.84, 1.51)
Suprapatellar effusion-synovitis	1.31 (0.96, 1.80)	1.15 (0.89, 1.48)
Central portion effusion-synovitis	1.14 (0.83, 1.58)	1.02 (0.75, 1.39)
Posterior femoral recess effusion-synovitis	1.10 (0.83, 1.46)	1.01 (0.76, 1.33)
Subpopliteal recess effusion-synovitis	1.22 (0.99, 1.52)	1.09 (0.88, 1.34)
IPFP quality score (0–6)	**1.40 (1.24, 1.57)**	**1.20 (1.06, 1.37)**
IPFP maximum area (cm^2^)	0.96 (0.77, 1.20)	0.96 (0.78, 1.19)
IPFP abnormal change (yes/no)	**3.17 (1.31, 7.65)**	1.87 (0.73, 4.79)
Increased lateral MRI-detected OP (yes or no)		
Total effusion-synovitis	**1.40 (1.13, 1.75)**	**1.27 (1.03, 1.57)**
Suprapatellar effusion-synovitis	**1.37 (1.22, 1.68)**	**1.22 (1.01, 1.47)**
Central portion effusion-synovitis	**1.24 (1.00, 1.53)**	1.16 (0.97, 1.40)
Posterior femoral recess effusion-synovitis	1.18 (0.97, 1.43)	1.08 (0.91, 1.28)
Subpopliteal recess effusion-synovitis	1.12 (0.96, 1.31)	1.08 (0.94, 1.25)
IPFP quality score (0–6)	**1.29 (1.19, 1.40)**	**1.19 (1.08, 1.30)**
IPFP maximum area (cm^2^)	1.02 (0.89, 1.18)	1.05 (0.91, 1.21)
IPFP abnormal change (yes/no)	**1.87 (1.15, 3.03)**	1.40 (0.87, 2.25)

Dependent variable: increases in magnetic resonance imaging (MRI)-detected osteophytes (OPs) (yes or no). Independent variables: baseline effusion-synovitis

*IPFP* intrapatellar fat pad

*Adjusted for age, sex and body mass index

**Further adjusted for bone marrow lesions, cartilage defects and baseline OPs. Significant differences are shown in bold

Baseline IPFP quality scores were significantly associated with increases in total MRI-detected OPs both before and after adjustment for covariates. In the medial tibiofemoral compartment, baseline IPFP quality score and presence of IPFP abnormality was significantly associated with increased MRI-detected OPs after adjustment for age, sex and BMI, but only IPFP quality score remained significant after further adjustments. IPFP quality scores and presence of IPFP abnormality were significantly associated with increased MRI-detected OPs in the lateral tibiofemoral compartment after adjustment for age, sex and BMI, but only IPFP quality scores persisted after further adjustment. IPFP maximum area was not associated with increased MRI-detected OPs in any compartments (Table 3).

## Discussion

To the best of our knowledge, this is the first study to describe the natural history of MRI-detected OPs and structural factors associated with this change. In this older adult sample, MRI-detected OPs were common, progressed in nearly half of participants over 2.6 years and rarely regressed. Baseline BMLs, cartilage defects, meniscal extrusion, presence of IPFP abnormality and effusion-synovitis were associated with worsening MRI-detected OPs, suggesting MRI-detected OPs are consequences of knee structural abnormalities.

Previous studies showed that knee MRI-detected OPs are far more common than OPs detected by conventional radiographs. One study reported that MRI-detected OPs were present in 60% of older persons without radiographic OA [32], and another found that the prevalence of MRI-detected OPs was 72% among middle-aged women [14]. Our current study is largely in line with these studies, with MRI-detected OPs present in 85% of a community-based older population. We also found that 51% of those participants who had MRI-detected OPs at baseline progressed over time, compared with only 19% of those with no MRI-detected OPs at baseline. Hart et al. investigated the natural history of grade-1 OPs measured by radiography in a 10-year follow-up knee study and reported that 62% of participants graded at baseline with a “doubtful” OP went on to develop confirmed radiographic knee OA compared with only 22% of controls with no sign of disease [33]. In our current cohort, 46% of all participants had increased total knee MRI-detected OPs over 2.6 years. Leyland et al. reported the annual cumulative incidence of radiographic knee OA was 2.3% between baseline and year 15, and participants with a Kellgren-Lawrence score of 1 were four times more likely to experience worsening by year 15 compared with participants with a baseline grade 0 [17]. These findings indicate that early OP formation on radiographs can be used as an early marker of initiation of disease process and when identified by primary healthcare provider, should warrant further action. It is reasonable to hypothesize that the same is true for MRI-detected OP based on this paper and our recent report [13]. The apparently higher rate of change in MRI-detected OPs raises the possibility of MRI being used instead of x-rays for monitoring OP progression.

Correlation between cartilage damage and MRI-defined OPs has been reported previously [34]. But few other studies have examined the associations of IPFP abnormality and effusion-synovitis with MRI-detected OP progression. One cross-sectional study suggested, unsurprisingly, that greater size of MRI-detected OPs related to severity of radiographic OA [32]. Another cross-sectional study revealed that MRI-detected OPs were weakly associated with synovitis or joint effusion but not correlated with Kellgren-Lawrence score [35]. Hill et al. reported that change in synovitis correlated with change in knee pain, but not loss of cartilage [36]. The only longitudinal study to be published revealed significant associations between MRI-detected OP volume and cartilage thickness loss but did not investigate associations with other structures [16]. In the current study, baseline BMLs, cartilage defects, meniscal extrusion, IPFP abnormality and effusion-synovitis were associated with worsening MRI-detected OPs over time, but presence of IPFP abnormality, IPFP maximum area, effusion-synovitis in the central portion, posterior femoral recess and subpopliteal recess were not independently associated with worsening MRI-detected OPs over time. Although the underlying structural mechanisms are largely unknown, these findings reinforce the evolving concept that knee OA is a whole-organ disease and that most structures are involved, including cartilage, meniscal, effusion-synovitis, subchondral bone and IPFP.

This study also found that participants with higher BMI had a higher rate of OP progression, which is in line with previous studies [37, 38]. The increased loading may have altered the biomechanics of the knee joints and accelerated the progression of OP, while a sedentary lifestyle may contribute to rapid progression of both BMI and OPs [39].

Although cartilage damage and OP formation are not perfectly correlated [40], joint space narrowing, which is a surrogate of cartilage damage, has been reported to be highly associated with the presence of OPs [41]. In animal models, OPs have been found to develop at sites of adjacent cartilage loss [42]. This is consistent with the current finding that cartilage defects at baseline site-specifically predicted an increase in MRI-detected OPs over 2.6 years.

In an older population sample, a fair proportion (61%) of the participants with meniscal abnormalities had no knee pain [43]. However, meniscal pathology is associated with the development of radiographic OA [44–46]. The existence of meniscal damage in a compartment appears to be a factor affecting the progression of OPs. Moreover, a study suggested that the mechanical stimuli in certain compartments may translate to factors on a cellular level or autonomous biochemical stimuli that initiate the process of OP formation [40, 47]. Felson et al. suggested that OPs are not directly involved in disease progression but might serve as markers of the location and severity of the pathologic process [8]. Our study found that BMLs, cartilage defects, meniscal extrusion, IPFP abnormality and effusion-synovitis were the structural risk factors for worsening MRI-detected OP, suggesting that MRI-detected OP could be a result of other osteoarthritic structural abnormalities. On the other hand, it should be noted that MRI-detected OPs were also found to consistently and independently predict changes in knee cartilage, BMLs and cartilage volume, and the need to undergo total knee replacement (TKA) [13, 15]. It is thought that subchondral bone expansion leads to splitting of cartilage and is potentially a precursor to formation of cartilage defects [22, 48]. Combined with our current findings, these studies indicate that OP formation is involved in the OA disease pathway and can be both a risk factor and a consequence of knee OA progression, suggesting the processes are not independent but are linked.

A combination of WORMS and KOSS for the measurement of OPs was employed in the present study. These two grading systems are validated instruments, which have good reliability to assess OPs semi-quantitatively on MRI [9, 20]. The WORMS grading system has the advantage of subdividing the whole knee into different subregions, which includes both marginal and central OPs, but its OP grading scale is more subjective. On the other hand, the KOSS grading system has the advantage of a quatitative OP grading scale for each subregion. The reliability of our measures was excellent.

Strengths of this study included the random selection of participants for the cohort from a community, with a large sample size. On the other hand, there are some potential limitations in our study. First, follow-up MRI scans were only available in 413 out of 837 participants due to decommissioning of the MRI scanner. However, there were no significant differences in demographic factors, ROA, baseline cartilage defects and BMLs between the current study sample and the rest of the cohort. Second, using a higher field-strength magnet than 1.5 T might be marginally more sensitive in detecting OPs; however, as reported previously [49], the results are unlikely to be markedly different as this benefit is modest. Third, the reproducibility for measurement of MRI-detected OPs was good rather than excellent, which may contribute to underestimation of the associations.

## Conclusion

Knee MRI-detected OPs are common in older adults and are likely to progress. The associations between baseline structural abnormalities and worsening MRI-detected OPs suggest MRI-detected OPs could be a consequence of multiple knee structural abnormalities.

## Abbreviations

BMI: Body mass index
BML: Bone marrow lesion
ICCs: Intraclass correlation coefficients
IPFP: Infrapatellar fat pad
KOSS: Knee Osteoarthritis Scoring System
MRI: Magnetic resonance imaging
OA: Osteoarthritis
OP: Osteophyte
ROA: Radiographic osteoarthritis
TASOAC: Tasmania Older Adult Cohort Study
TKA: Total knee arthroplasty
WORMS: Whole-Organ Magnetic Resonance Imaging Score
